# Comparative effects of six rehabilitation therapies on lower limb function and gait function in stroke patients: a network meta-analysis of 33 RCTs

**DOI:** 10.3389/fneur.2026.1759251

**Published:** 2026-03-06

**Authors:** Shuxin Zhang, Hongying Zhang, Zhaobin Miao, Jia Han

**Affiliations:** 1 School of Exercise and Health, Shanghai University of Sport, Shanghai, China; 2 Department of Rehabilitation, School of International Medical Technology, Shanghai Sanda University, Shanghai, China; 3 Department of Rehabilitation Medicine, Shanghai Second People's Hospital, Shanghai, China; 4 Department of Rehabilitation Medicine, Kong Jiang Hospital of Yangpu District, Shanghai, China

**Keywords:** gait function, lower limb function, network meta-analysis, rehabilitation intervention comparison, stroke rehabilitation

## Abstract

**Objective:**

This study aims to systematically compare the relative efficacy of six non-invasive rehabilitation interventions—Standardized Rehabilitation (SR), Aerobic Rehabilitation (AR), Resistance Training Rehabilitation (RTR), Intelligent Rehabilitation (IR), Traditional Chinese Rehabilitation (TCR), and Neuromodulation Rehabilitation (NR)—in improving lower limb function and gait function in stroke patients using a network meta-analysis approach. It also ranks the efficacy of each intervention.

**Methods:**

We systematically searched five databases—PubMed, Embase, EBSCO, Web of Science, and Scopus—for randomized controlled trials (RCTs) published from January 2003 to November 2025. Two researchers independently screened studies, extracted data, and assessed risk of bias. Stata 18.0 software was used for statistical analysis. Standardized mean differences (SMDs) and their 95% confidence intervals (CIs) were calculated. Probabilistic ranking of intervention efficacy was performed using the cumulative ordered ranking curve area (SUCRA) value.

**Results:**

A total of 33 RCTs were included. Network meta-analysis revealed: (1) For lower limb function improvement (using the Fugl-Meyer lower limb score as the core indicator), the efficacy ranking was IR (SUCRA = 75.7) > AR (60.7) > RTR (53.1) > SR (10.5). Smart rehabilitation had the highest probability (46.7%) of being the optimal approach. (2) For gait function improvement (core measures: walking speed, 6-min walk test), the efficacy ranking was NR (SUCRA = 82.2) > IR (71.6) > AR (50.8) > RTR (38.8) > TCR (35.7) > SR (20.9). Neuromodulation rehabilitation had the highest probability of being the optimal solution (36.5%). Direct and indirect comparison results were largely consistent, with funnel plots showing no significant publication bias.

**Conclusion:**

Based on existing evidence, intelligent rehabilitation may offer relative advantages in improving lower limb function in stroke patients, while neuromodulation rehabilitation demonstrates greater potential for enhancing gait function. Standardized rehabilitation, as a conventional baseline approach, demonstrated relatively weaker effects. The ranking results from this study provide evidence-based guidance for clinicians selecting individualized rehabilitation programs targeting different functional goals. Future high-quality research is needed to validate and refine intervention recommendations for different disease stages.

## 1 Introduction

Stroke is a highly prevalent cerebrovascular disease worldwide and has become the leading cause of disability in adults, with both incidence and disability rates remaining persistently high (1). According to the World Health Organization, over 15 million new stroke cases occur globally each year, with China accounting for nearly 40% of these cases. Moreover, the incidence rate continues to increase at an annual rate of 8.7% (2). Following stroke, lower limb and gait dysfunction represent one of the most common sequelae, occurring in 70%−80% of cases. Primary manifestations include decreased lower limb muscle strength, abnormal muscle tone, impaired balance, slowed walking speed, and gait instability (3, 4). These impairments severely limit patients' ability to live independently, hindering daily activities like walking, stair climbing, dressing, and personal hygiene. This not only significantly reduces quality of life but also imposes heavy caregiving burdens and financial pressures on families (5). From a public health perspective, the long-term care needs of a large number of stroke-disabled patients further exacerbate the consumption of medical resources, presenting a major public health challenge that requires urgent attention (6).

There exists a distinct critical window period for the recovery of lower limb and gait function following stroke. During this phase, neural plasticity is heightened, and timely, standardized rehabilitation interventions can effectively promote neural functional remodeling and improve motor outcomes. The restoration of lower limb and gait function serves as a crucial foundation for patients to regain independent mobility and reintegrate into family and societal life. Consequently, rehabilitation holds an indispensable position within the comprehensive management of stroke (7). Currently, rehabilitation interventions for lower limb and gait function in stroke patients are increasingly diverse, primarily encompassing six core approaches: Standardized rehabilitation, grounded in evidence-based practice, establishes a unified protocol covering joint range of motion, balance, and gait training, serving as a commonly used clinical routine (8); aerobic rehabilitation enhances cardiopulmonary function and endurance through sustained low-to-moderate intensity exercise, providing physical support for lower limb functional recovery (9); resistance rehabilitation employs elastic bands, sandbags, or weighted equipment for targeted muscle strength training, enhancing hip, knee, and ankle muscle groups to improve movement control and gait stability (10). Intelligent rehabilitation leverages technologies like rehabilitation robots and virtual reality, utilizing precise feedback, repetitive training, and scenario simulation to enhance the scientific rigor and engagement of rehabilitation, suitable for patients at varying functional levels (11–13); Traditional Chinese rehabilitation, guided by TCM theory, incorporates methods such as acupuncture, tuina massage, and traditional qigong to regulate qi and blood, unblock meridians, and improve local circulation and neural function (14, 15). Neurostimulation rehabilitation employs techniques like transcranial magnetic stimulation and mirror neuron therapy to directly modulate the excitability of relevant neural pathways, promoting recovery of impaired motor function (16, 17).

Despite the abundance of rehabilitation interventions, existing studies are largely confined to direct comparisons between single interventions and either standard care or another intervention, lacking comprehensive cross-sectional comparisons among different measures. This makes it difficult for clinicians to assess the relative efficacy of various interventions, select the optimal approach based on patient-specific conditions, or provide evidence-based support for ranking measures in clinical guidelines. Network meta-analysis can simultaneously integrate evidence from direct and indirect comparisons, construct intervention networks, quantify the relative efficacy between different measures, and rank multiple rehabilitation protocols. This approach provides more comprehensive, tiered evidence for clinical decision-making, addressing current research gaps (18). Based on this, this study employs network meta-analysis to systematically evaluate the differential effects of the aforementioned six rehabilitation measures on lower limb function and gait function in stroke patients. The study aims to identify which intervention yields the most significant improvement in lower limb motor function, which intervention provides the optimal enhancement in gait function, and the relative efficacy ranking of the six interventions in improving lower limb and gait function. The findings will provide high-quality evidence for developing individualized rehabilitation plans in clinical practice and serve as a reference for guideline development and future research directions in stroke rehabilitation.

## 2 Methods

This study adheres to the PRISMA (Preferred Reporting Items for Systematic Reviews and Meta-Analyses) guidelines and the standards outlined in the Cochrane Handbook for Systematic Reviews. The study protocol has been registered with the International Prospective Systematic Reviews Register (PROSPERO), with the registration number CRD420251245022.

### 2.1 Data sources and retrieval strategy

A total of five databases—PubMed, Embase, EBSCO, Web of Science, and Scopus—were searched. To ensure comprehensiveness and precision, the PubMed database was used as an example to illustrate the combined search strategy of “MeSH Terms + free-text terms,” with search strings constructed according to the PICOS principle. For the subject dimension, MeSH terms such as “Stroke” and “Cerebrovascular Accident” were combined with free terms like “stroke” and “apoplexy” to cover ischemic and hemorrhagic strokes along with various synonymous expressions. For the intervention dimension, MeSH terms including “Rehabilitation” and “Physical Therapy Modalities” were integrated with free terms such as “exercise therapy” and “gait training,” comprehensively covering six core types of rehabilitation interventions. For the outcome dimension, MeSH terms like “Lower Extremity” and “Gait” were used alongside free terms such as “gait assessment” and “gait analysis,” ensuring thorough coverage of six core outcome categories. Physical therapy modalities and free terms like “exercise therapy” and “gait training” “to comprehensively cover six core rehabilitation intervention types. For outcome measures, terms like” Lower Extremity “and “Gait” combined with free terms such as” lower extremity function “and” “walking ability” precisely targeted studies related to lower limb function and gait function. For study design, the search was restricted to the subject term “Randomized Controlled Trials as Topic” and the document type “Randomized Controlled Trial” to ensure inclusion of high-quality direct comparative studies, providing reliable data support for the network meta-analysis. The complete search query is as follows: ((“Stroke”[MeSH Terms] OR “Cerebrovascular Accident”[MeSH Terms] OR “Cerebral Infarction”[MeSH Terms] OR “Cerebral Hemorrhage”[MeSH Terms] OR stroke OR cerebrovascular accident OR cerebral infarction OR cerebral hemorrhage OR apoplexy) AND (“Rehabilitation”[MeSH Terms] OR “Physical Therapy Modalities”[MeSH Terms] OR “Occupational Therapy”[MeSH Terms] OR “Exercise Therapy”[MeSH Terms] OR rehabilitation OR physical therapy OR occupational therapy OR exercise therapy OR balance training OR gait training) AND (“Lower Extremity” [MeSH Terms] OR “Motor Function”[MeSH Terms] OR “Gait”[MeSH Terms] OR “Gait Disorders, Neurologic”[MeSH Terms] OR lower extremity function OR motor function OR gait function OR ambulation OR walking ability) AND (“Randomized Controlled Trials as Topic”[MeSH Terms] OR “Randomized Controlled Trial”[ptyp])).

### 2.2 Study selection and inclusion criteria

Inclusion criteria were established based on the PICOS framework:

P: Adult patients with a confirmed diagnosis of stroke, regardless of disease stage, stroke type, or severity.
I: Six non-invasive rehabilitation therapies. Specifically including standardized rehabilitation, aerobic rehabilitation, resistance rehabilitation, smart rehabilitation, traditional Chinese rehabilitation, and neuromodulation rehabilitation.
C: Standardized rehabilitation or any other active non-invasive therapy.
O: Studies must report primary outcomes in at least one of the following dimensions: lower limb function using the lower limb section of the FMA scale; gait function reported via step rate, 6-min walk test, or 10-m walk test.
S: Randomized controlled trials.

Studies involving stroke patients with systemic comorbidities, non-randomized controlled trials, animal experiments, reviews, conference abstracts, case reports, studies with unobtainable data, or studies where complete data remained unavailable after contacting authors will be excluded.

### 2.3 Data extraction

Literature screening and data extraction were performed independently by two researchers. First, duplicate records were removed using EndNote X9 software (Clarivate Analytics, Philadelphia, PA, United States). Initial screening was conducted by reviewing titles and abstracts, followed by full-text reading of remaining studies to determine final inclusion. The two researchers cross-checked their screening results; disagreements were resolved through discussion or consultation with a third researcher. Data extraction was performed using a pre-designed standardized form, capturing: study details (first author, publication year, country); participant characteristics (sample size per group, age, gender, and post-stroke duration); detailed intervention measures (specific therapy type, treatment frequency, single session duration, and total treatment course); and outcome measure data (mean values, standard deviations, or corresponding change values before and after treatment).

### 2.4 Risk of bias in included studies

Two researchers (Shuxin Zhang and Hongying Zhang) independently assessed risk of bias using the Cochrane 5.1 risk of bias tool across seven domains: random sequence generation, allocation concealment, blinding of patients and therapists, blinding of outcome assessors, incomplete outcome data, selective reporting of results, and other sources of bias. Risk assessments were subsequently analyzed using Review Manager 5.3 (Cochrane, Copenhagen, Denmark), categorizing each domain as “low risk,” “high risk,” or “unclear.” Final assessments were cross-checked by both researchers, with any discrepancies resolved through discussion or consultation with a third researcher to determine study eligibility.

### 2.5 Statistical analysis

This study employed Stata 18.0 software (StataCorp LLC, College Station, TX, United States) for meta-analysis, with outcome measures as continuous variables. The network meta-analysis (NMA) integrated pre- and post-intervention changes in both treatment and control groups to systematically evaluate the effects of different rehabilitation interventions on lower limb function and gait parameters in stroke patients. To accurately assess the effectiveness of these interventions, standardized mean differences (SMDs) and their 95% confidence intervals (CIs) were calculated for each outcome measure. A uniform significance level of α = 0.05 was set, and a random-effects model was employed for pooled effect size estimation to account for heterogeneity among studies regarding participant characteristics and intervention modalities. Heterogeneity was quantified using the *I*^2^ statistic and Cochran's *Q*-test. Network diagrams visualized relationships among different non-invasive methods, where connections between nodes represented direct comparisons. Node size and line thickness were proportional to the number of studies included in each comparison, enabling intuitive display of relative strengths and positioning within the network. Furthermore, network contribution plots were generated to quantify each direct comparison's contribution to the overall network, aiding analysis of interventions' impact within the network. To assess publication bias, corrected comparison funnel plots analyzed publication bias for primary outcome measures. Finally, the cumulative ranking curve under the area (SUCRA) method was used to calculate the probability of each intervention being the optimal treatment.

## 3 Results

### 3.1 Literature screening results

**Figure 1:**
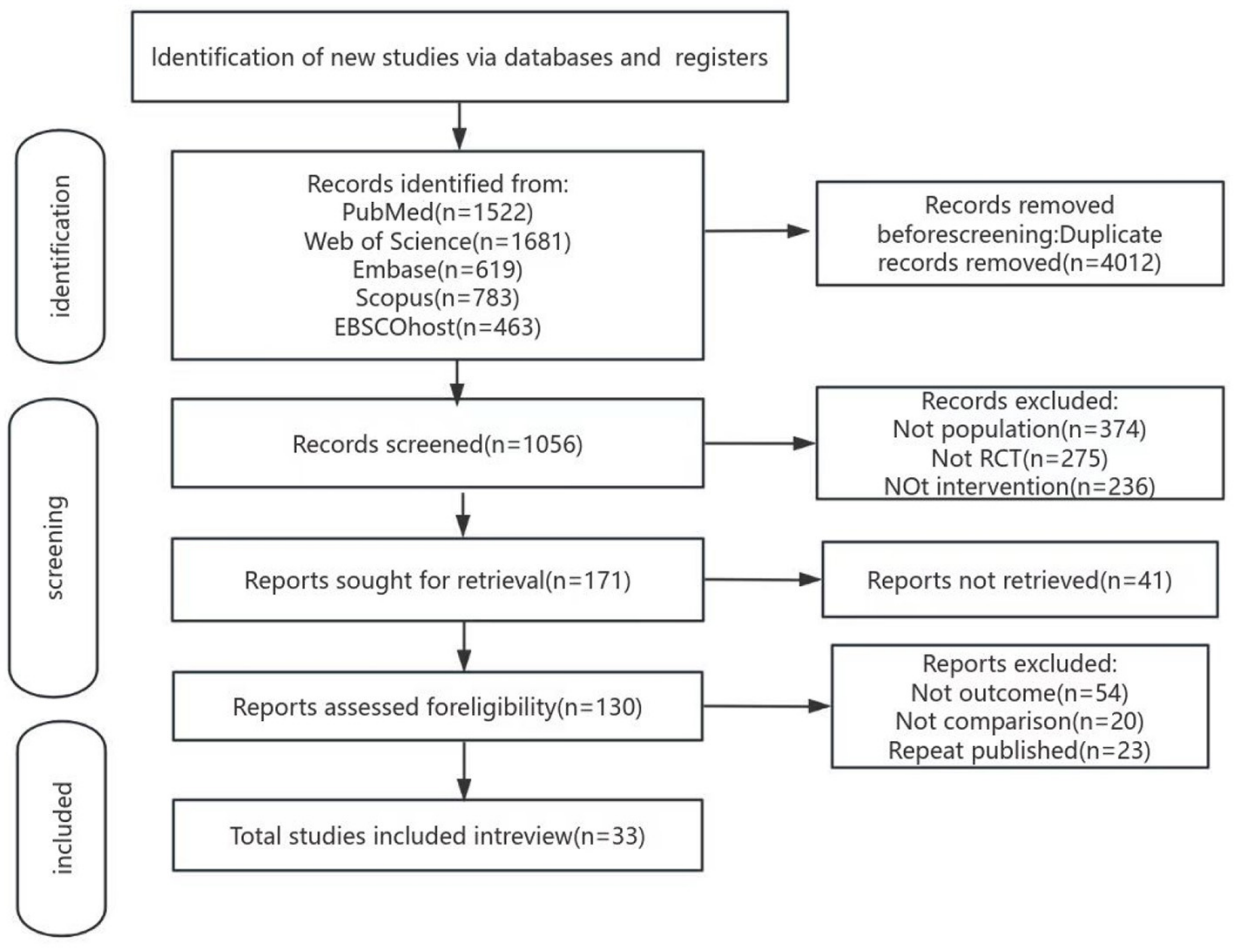
Literature search flowchart.

The literature screening flowchart is shown in Figure 1. A total of 5,068 potentially eligible studies were retrieved from five databases. To ensure study accuracy and avoid duplicate counting of identical content, 4,012 duplicate records were removed through automated and manual checks, leaving 1,056 studies for screening. By analyzing the titles and abstracts of each article, 885 papers that did not meet the inclusion criteria were excluded, retaining only those most relevant to the study objectives. Full texts of 130 articles were obtained and reviewed, with detailed assessments of study design, sample size, methodological quality, and outcomes. Ultimately, 33 randomized controlled trials meeting the study's quality criteria were identified, evaluating six distinct rehabilitation intervention formats. Each step of the screening process adhered to a strictly standardized protocol to ensure the reliability and scientific rigor of the results.

### 3.2 Basic characteristics included in the study

The basic characteristics of the 33 included RCTs are detailed in Supplementary Appendix 1. These studies were published between 2009 and 2025 and conducted across multiple countries worldwide, including India, Brazil, the USA, Korea, China, Pakistan, Hungary, Canada, the UK, Norway, Cleveland, and Saudi Arabia. A total of 1,907 stroke patients were involved, covering various disease stages from the acute to chronic phases. Regarding interventions, the network encompassed all six predefined rehabilitation therapies. Treatment durations ranged from 2 weeks to 6 months. The basic characteristics of the included studies are detailed in Supplementary Appendix 1.

### 3.3 Bias risk assessment

**Figure 2:**
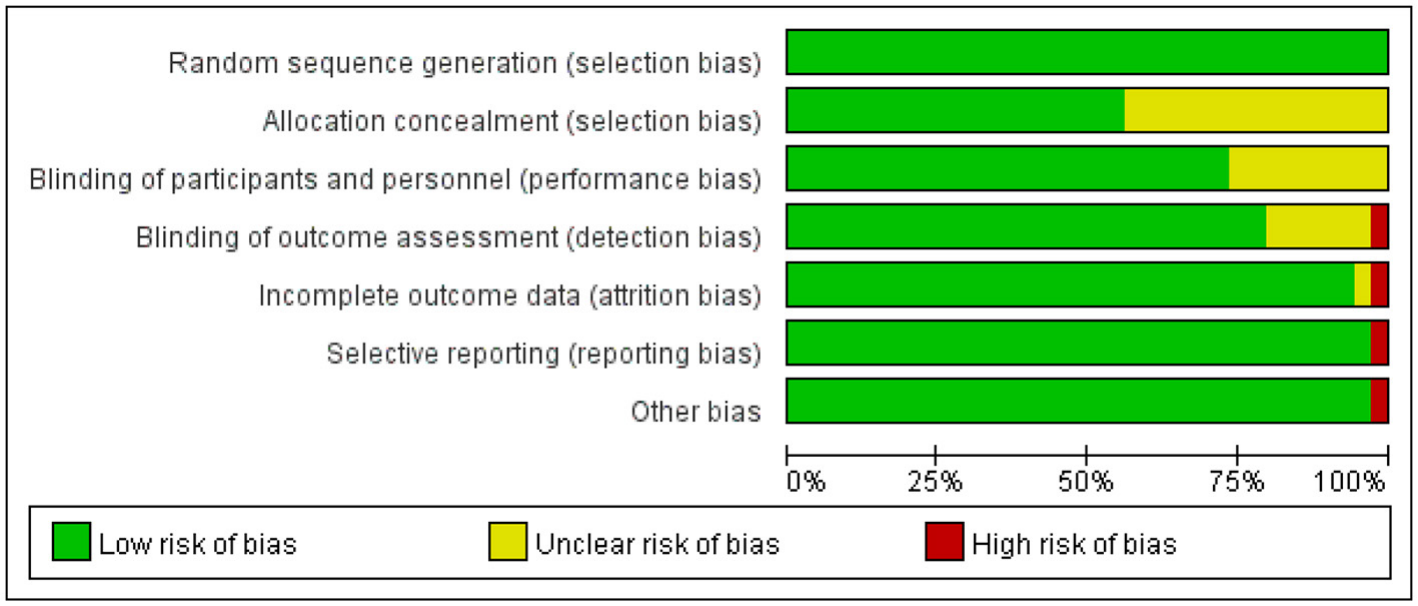
Risk of bias in studies included in this meta-analysis.

**Figure 3:**
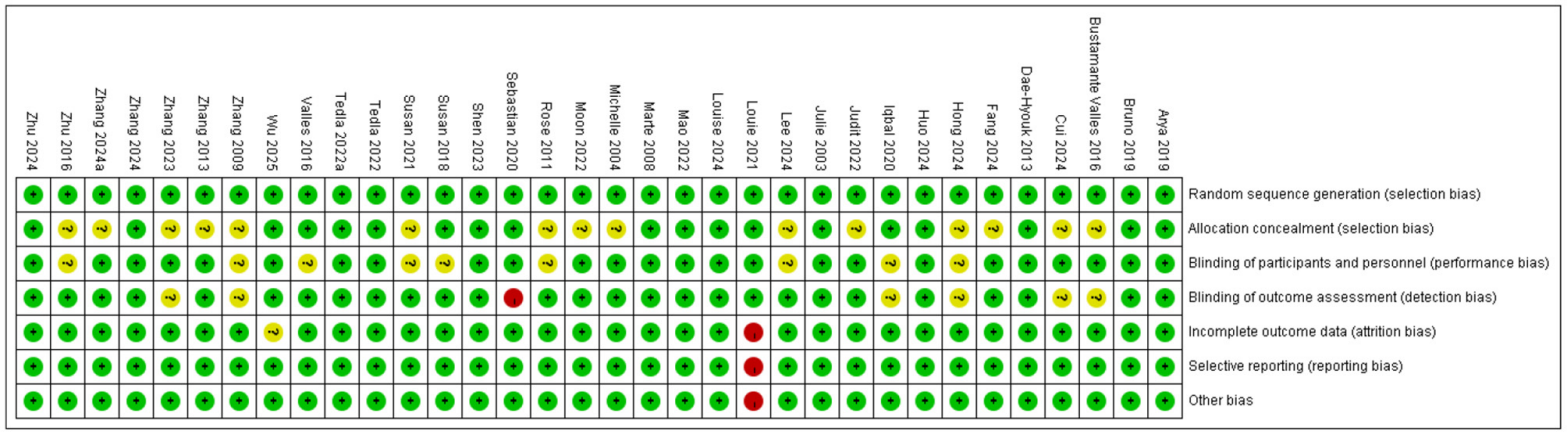
Evaluation results for each methodological quality item included in the study.

According to the Cochrane ROB 2.0 tool (Cochrane, London, United Kingdom) assessment, studies included in this systematic review generally demonstrated good methodological quality in random sequence generation, with the vast majority rated as low risk. Regarding allocation concealment and blinding of outcome assessors, approximately half of the studies provided adequate methodological descriptions and met low-risk criteria, while the remaining studies were rated as uncertain risk due to unclear reporting of information. Due to the unique nature of rehabilitation interventions, most studies faced challenges in blinding patients and therapists, resulting in a high risk of bias prevalent in this field. Regarding data integrity, incomplete outcome data and selective reporting of results were rated as low risk in the majority of studies. Notably, a few studies were rated as high risk due to baseline imbalance between groups. Overall, the risk of bias in the included studies was acceptable, predominantly low or moderate risk. (See Table 1 and Figures 2, 3).

**Table 1:** Risk of bias assessment results for included studies.

**Study**	**Random sequence generation**	**Allocationconcealment**	**Blinding of participants and personnel**	**Blinding of outcome assessment**	**Incomplete outcome data**	**Selective reporting**	**Otherbias**
Arya 2019	L	L	L	L	L	L	L
Bruno 2019	L	L	L	L	L	L	L
Bustamante Valles 2016	L	U	L	U	L	L	L
Cui 2024	L	U	L	U	L	L	L
Dae-Hyouk 2013	L	L	L	L	L	L	L
Fang 2024	L	U	L	L	L	L	L
Hong 2024	L	U	U	U	L	L	L
Huo 2024	L	L	L	L	L	L	L
Iqbal 2020	L	L	U	U	L	L	L
Judit 2022	L	U	L	L	L	L	L
Julie 2003	L	L	L	L	L	L	L
Lee 2024	L	U	U	L	L	L	L
Louie 2021	L	L	L	L	H	H	H
Louise 2024	L	L	L	L	L	L	L
Mao 2022	L	L	L	L	L	L	L
Marte 2008	L	L	L	L	L	L	L
Michelle 2004	L	U	L	L	L	L	L
Moon 2022	L	U	L	L	L	L	L
Rose 2011	L	U	U	L	L	L	L
Sebastian 2020	L	L	L	H	L	L	L
Shen 2023	L	L	L	L	L	L	L
Susan 2018	L	L	U	L	L	L	L
Susan 2021	L	U	U	L	L	L	L
Tedla 2022	L	L	L	L	L	L	L
Valles 2016	L	L	U	L	L	L	L
Wu 2025	L	L	L	L	L	L	L
Zhang 2009	L	U	U	U	L	L	L
Zhang 2013	L	U	L	L	L	L	L
Zhang 2023	L	U	L	U	L	L	L
Zhang 2024	L	L	L	L	L	L	L
Zhang 2024	L	U	L	L	L	L	L
Zhu 2016	L	U	U	L	L	L	L
Zhu 2024	L	L	L	L	L	L	L

### 3.4 Direct pairwise meta-analysis

**Figure 4:**
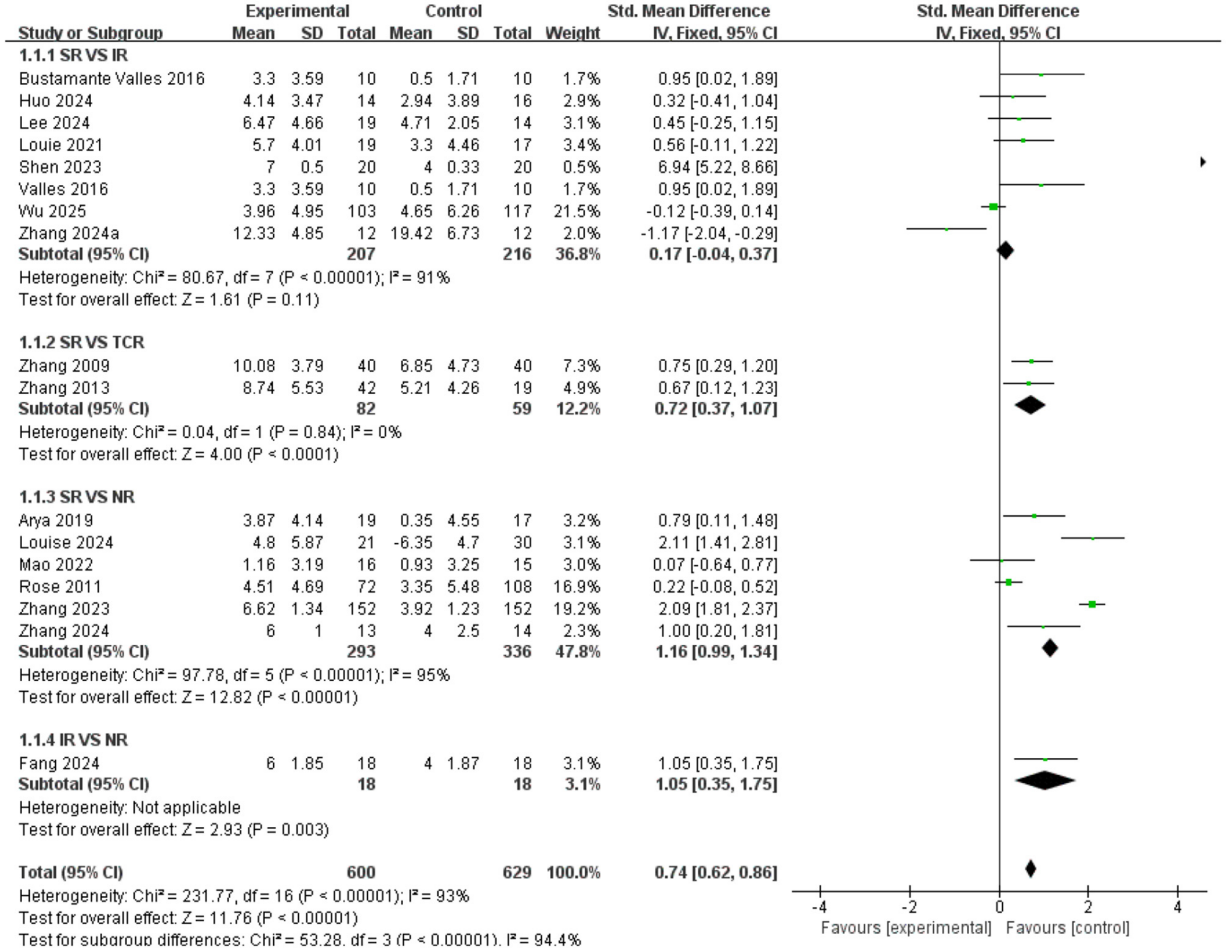
Forest plot of lower limb function; standardized mean differences (SMDs) for each individual study are represented by squares, with size reflecting statistical weight; horizontal lines denote 95% confidence intervals (CI). Diamonds represent pooled SMDs for each subgroup and the overall analysis.

Compared with standardized rehabilitation (SR), intelligent rehabilitation (IR) did not demonstrate a significant advantage in improving lower limb function [SMD = 0.17, 95% CI (−0.04, 0.37), *P* = 0.11], but high heterogeneity existed among studies (*I*^2^ = 91%), suggesting substantial variability in the effectiveness of this intervention across different research settings. Traditional Chinese medicine rehabilitation (TCR) demonstrated significant efficacy [SMD = 0.72, 95% CI (0.37, 1.07), *P* < 0.0001) with high consistency across studies (*I*^2^ = 0%); Neurostimulation rehabilitation (NR) also demonstrated significant effects [SMD = 1.16, 95% CI (0.99, 1.34), *P* < 0.0001]. Although high heterogeneity existed among studies (*I*^2^ = 95%), the effect direction was consistent. Direct comparisons revealed that intelligent rehabilitation (IR) demonstrated a significant advantage over neuromodulation rehabilitation [NR; SMD = 1.05, 95% CI (0.35, 1.75), *P* = 0.003], providing crucial evidence for future intervention selection (see Figure 4).

**Figure 5:**
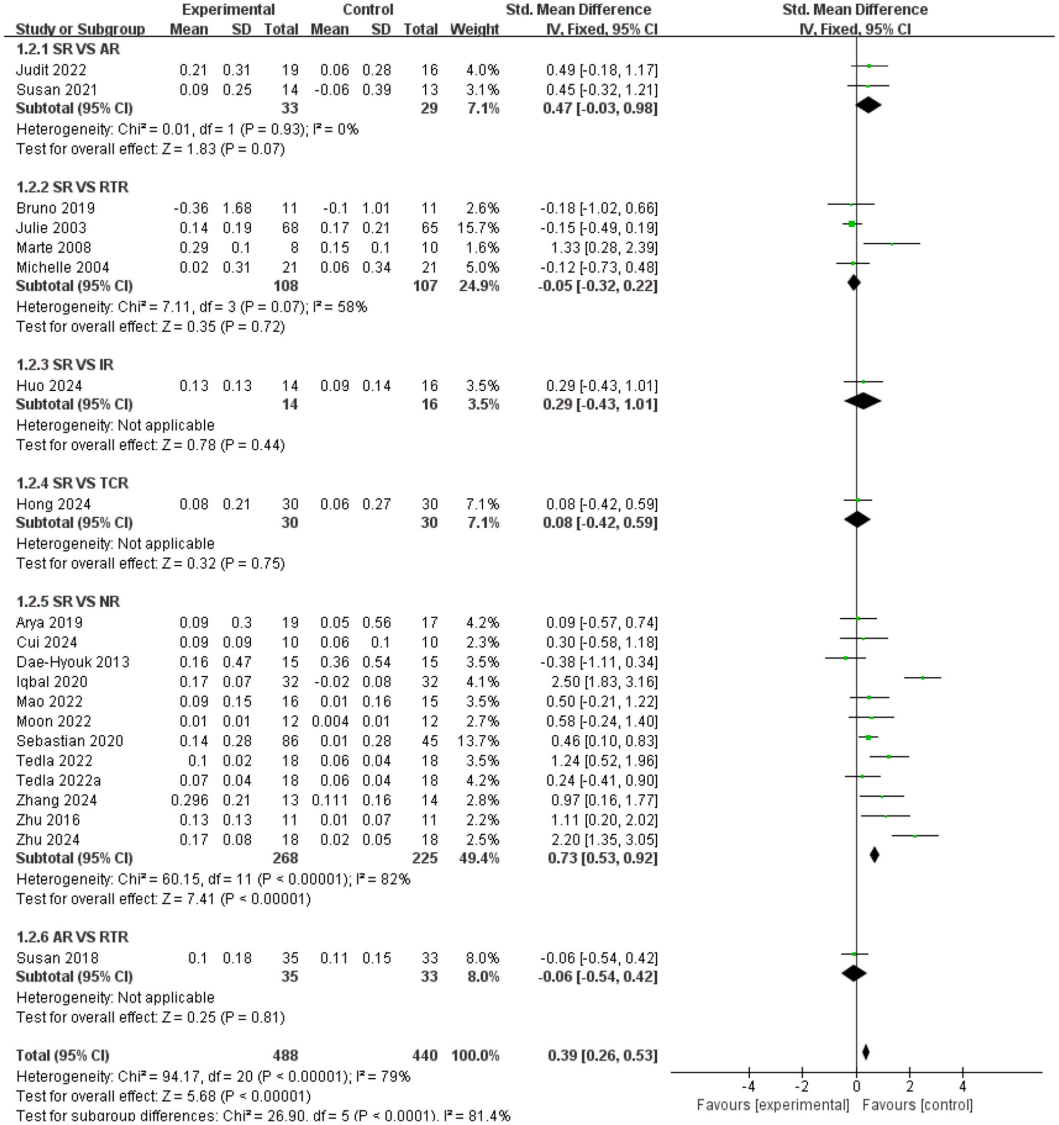
Forest plot of gait function; standardized mean differences (SMDs) for each individual study are represented by squares, with size reflecting statistical weight; horizontal lines denote 95% confidence intervals (CI). Diamonds represent pooled SMDs for each subgroup and the overall analysis.

Results of pairwise comparisons for gait function measures are shown in Figure 5. Compared with standardized rehabilitation (SR), aerobic rehabilitation (AR) showed no significant advantage [SMD = 0.47, 95% CI (−0.03, 0.98), *P* = 0.93], but with low study heterogeneity (*I*^2^ = 0%); Resistance training rehabilitation (RTR) also showed no significant difference [SMD = −0.05, 95% CI (−0.32, 0.22), *P* = 0.07], with moderate heterogeneity among studies (*I*^2^ = 58%); The effect of intelligent rehabilitation (IR) also failed to reach statistical significance [SMD = 0.29, 95% CI (−0.43, 1.01), *P* = 0.44]; Traditional Chinese medicine rehabilitation (TCR) showed no significant difference compared to SR [SMD = 0.08, 95% CI (−0.42, 0.59), *P* = 0.75]. Notably, neuromodulation rehabilitation (NR) demonstrated significantly superior outcomes compared to SR [SMD = 0.73, 95% CI (0.53, 0.92), *P* < 0.00001], despite high heterogeneity among studies (*I*^2^ = 82%). In direct comparisons, no significant difference was observed between aerobic rehabilitation (AR) and resistance training rehabilitation [RTR; SMD = −0.06, 95% CI (−0.54, 0.42), *P* = 0.81; see Figure 5].

### 3.5 Network meta-analysis

#### 3.5.1 Network diagram of included studies

**Figure 6:**
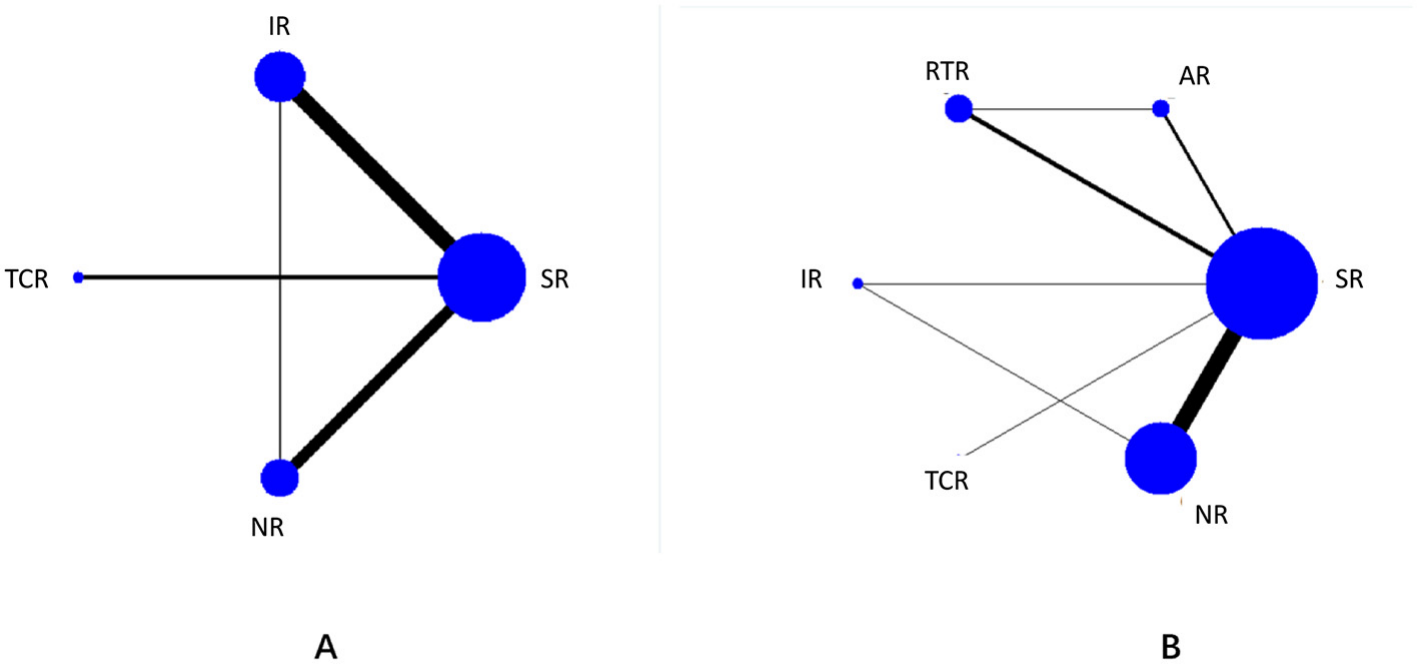
Network diagram: **(A)** represents lower limb function; **(B)** represents gait function. 1 = SR; 2 = AR; 3 = RTR; 4 = IR; 5 = TCR; 6 = NR.

The seven nodes in the figure represent seven intervention measures. Straight lines between nodes indicate direct comparisons between interventions, with line thickness reflecting the number of studies comparing the two interventions. Among these, SR is the most extensively studied intervention, while research on TCR is relatively scarce. The outcome indicator network diagram is detailed in Figure 6.

#### 3.5.2 Ranking of intervention effects for six different exercise modalities

**Figure 7:**
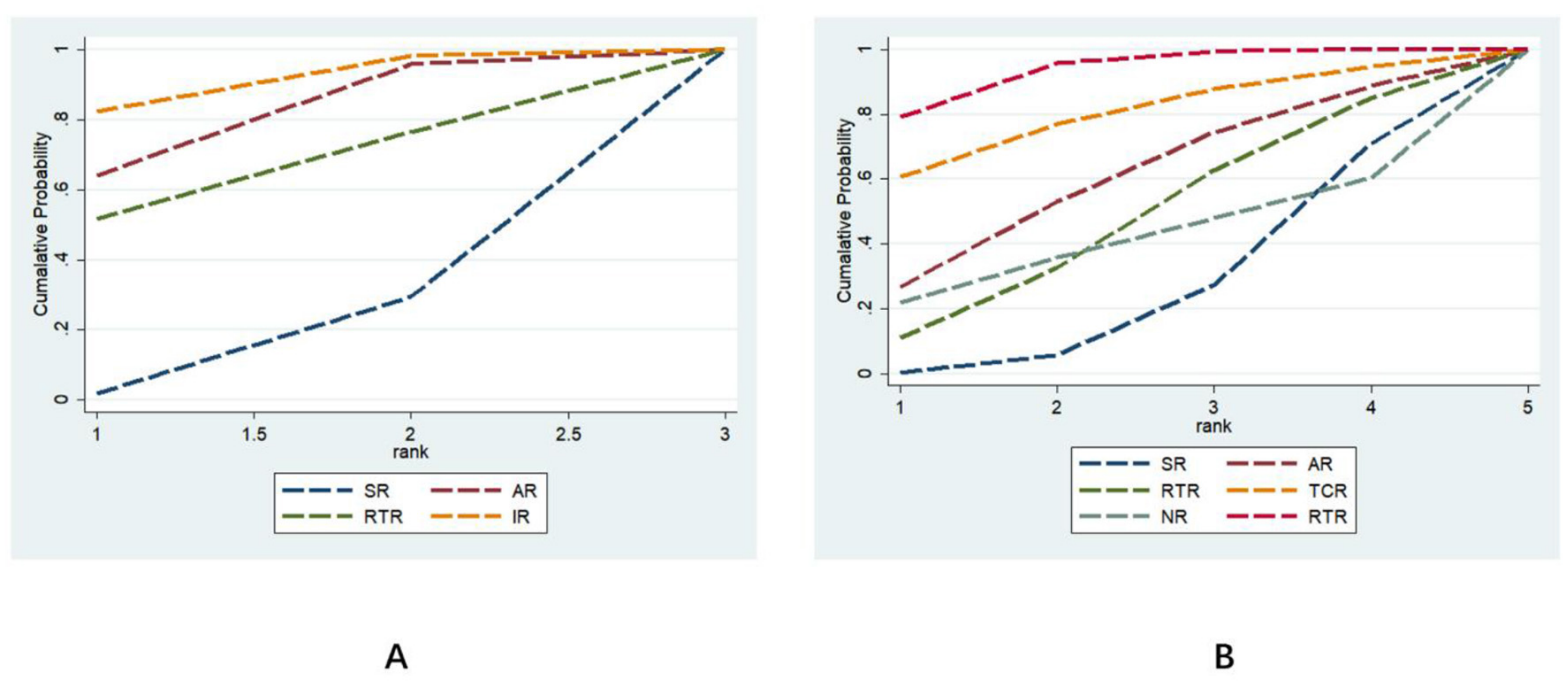
Effectiveness ranking chart for outcome measures. **(A)** represents lower limb function; **(B)** represents gait function.

Lower Limb Functional Indicators: The effectiveness ranking of six different rehabilitation intervention models in improving lower limb function in stroke patients was: IR (SUCRA = 75.7) > AR (SUCRA = 60.7) > RTR (SUCRA = 53.1) > SR (SUCRA = 10.5). Intelligent rehabilitation (IR) had the highest probability of being the optimal intervention (PrBest = 46.7%). Aerobic Rehabilitation (AR) and Resistance Training Rehabilitation (RTR) had probabilities of 22.2 and 31.0%, respectively to be the best intervention, while Standardized Rehabilitation (SR) showed the weakest effect (PrBest = 0.1%). See Figure 7 and Table 2 for details. For sensitivity analysis regarding lower limb function, please refer to Supplementary Appendix 2.

**Table 2:** Ranking of six rehabilitation interventions for improving lower limb function and gait function in stroke patients.

**Intervention methods**	**Lower limb function**		**Gait function**	
	**SUCRA (%)**	**Ranking**	**SUCRA (%)**	**Ranking**
SR	10.5	4	20.9	6
AR	60.7	2	50.8	3
RTR	53.1	3	38.8	4
IR	75.7	1	71.6	2
TCR	/	/	35.7	5
NR	/	/	82.2	1

Gait Function Metrics: The effectiveness ranking of the six rehabilitation intervention modes in improving stroke patients' gait was: NR (SUCRA = 82.2) > IR (SUCRA = 71.6) > AR (SUCRA = 50.8) > RTR (SUCRA = 38.8) > TCR (SUCRA = 35.7) > SR (SUCRA = 20.9). Among these, neuromodulation rehabilitation (NR) had the highest probability of being the optimal intervention (PrBest = 36.5%), followed by intelligent rehabilitation (IR) at 38.0%, while standardized rehabilitation (SR) demonstrated the weakest efficacy (PrBest = 0.0%). See Figure 7 and Table 2 for details. For sensitivity analysis regarding gait function, please refer to Supplementary Appendix 2.

#### 3.5.3 Estimation of pooled effect sizes for primary outcome measures

Lower limb function: Compared with SR, IR [SMD = 0.17, 95% CI (−0.04, 0.37)], traditional Chinese medicine rehabilitation [TCR; SMD = 0.72, 95% CI (0.37, 1.07)], and NR [SMD = 1.16, 95% CI (0.99, 1.34)] all demonstrated positive effects in improving lower limb function in stroke patients. RTR [SMD = 0.71, 95% CI (−1.41, 2.83)] showed some improvement but no statistically significant difference. Detailed results for lower limb function are presented in Table 3.

**Table 3:** Network meta-analysis matrix for outcome measures.

**Lower limb function**					
IR	−0.31 (−1.76,1.14)	−0.46 (−2.88,1.95)	−1.17 (−2.32, −0.02)		
0.31 (−1.14,1.76)	AR	−0.15 (−2.52, 2.2 2)	−0.86 (−1.90, 0.18)		
0.46 (−1.95,2.88)	0.15 (−2.22, 2.5 2)	RTR	−0.71 (−2.83,1.41)		
1.17 (0.02,2.32)	0.86 (−0.18,1.90)	0.71 (−1.41, 2.83)	SR		
**Gait function**					
NR	−0.08 (−1.13, 0.96)	−0.42 (−1.37, 0.53)	−0.57 (−1.36, 0.22)	−0.69 (−2.12, 0.74)	−0.77 (−1.18, −0.36)
0.08 (−0.96,1.13)	IR	−0.34 (−1.69, 1.02)	−0.48 (−1.73,0.76)	−0.60 (−2.33,1.12)	−0.69 (−1.73, 0.36)
0.42 (−0.53,1.37)	0.34 (−1.02,1.69)	AR	−0.15 (−1.07,0.78)	−0.27 (−1.88, 1.35)	−0.35 (−1.21,0.51)
0.57 (−0.22,1.36)	0.48 (−0.76,1.73)	0.15 (−0.78,1.07)	RTR	−0.12 (−1.64,1.40)	−0.20 (−0.87, 0.47)
0.69 (−0.74,2.12)	0.60 (−1.12,2.33)	0.27 (−1.35, 1.88)	0.12 (−1.40,1.64)	TCR	−0.08 (−1.45,1.29)
0.77 (0.36,1.18)	0.69 (−0.36,1.73)	0.35 (−0.51,1.21)	0.20 (−0.47, 0.87)	0.08 (−1.29,1.45)	SR

Gait Function: Compared with SR, NR [SMD = 0.73, 95% CI (0.53, 0.92)] significantly improved gait in stroke patients. IR [SMD = 0.69, 95% CI (−0.36, 1.73)], AR [SMD = 0.35, 95% CI (−0.51, 1.21)], RTR [SMD = 0.20, 95% CI (−0.47, 0.87)], TCR [SMD = 0.08, 95% CI (−1.29, 1.45)] showed some improvement but without significant effect. Detailed gait-related results are presented in Table 3.

### 3.6 Small-sample effects or publication bias tests

**Figure 8:**
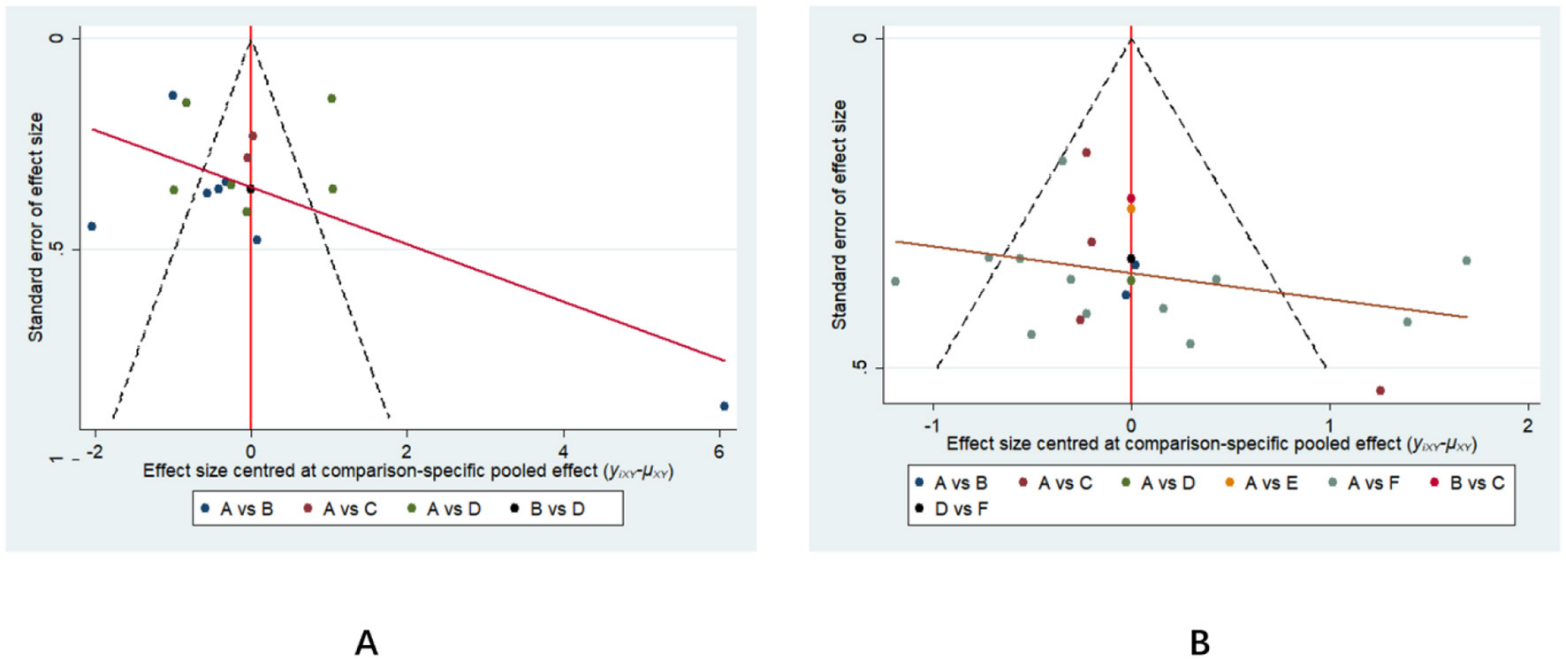
Outcome-adjusted comparison funnel plot. **(A)** represents lower limb function; **(B)** represents gait function. A = SR; B = AR; C = RTR; D = IR; E = TCR; F = NR.

A corrected funnel plot was employed to assess small-sample effects and examine publication bias in studies included in the network meta-analysis. Results indicated that the funnel plots for the included studies were largely symmetrical, suggesting no small-sample effects were present in this study. No significant publication bias was detected, as detailed in Figure 8.

## 4 Discussion

This study employed a network meta-analysis approach to systematically compare and rank the efficacy of six rehabilitation interventions—Standardized Rehabilitation (SR), Aerobic Rehabilitation (AR), Resistance Training Rehabilitation (RTR), Intelligent Rehabilitation (IR), Traditional Chinese Rehabilitation (TCR), and Neuromodulation Rehabilitation (NR)—in improving lower limb and gait function among stroke patients. Results revealed varying efficacy among rehabilitation interventions, with distinct ranking patterns observed for lower limb and gait function. Overall, in terms of lower limb function improvement, Intelligent Rehabilitation (IR) demonstrated the greatest potential as the optimal intervention, followed by Aerobic Rehabilitation (AR) and Resistance Training Rehabilitation (RTR), while Standardized Rehabilitation (SR) showed the weakest effect. For gait function improvement, neuromodulation rehabilitation (NR) ranked highest (SUCRA = 82.2), followed by intelligent rehabilitation (IR), with standardized rehabilitation (SR) again ranking last. This indicates that interventions integrating technology-assisted methods (e.g., robotics, virtual reality) or neuromodulation techniques, when applied on top of conventional rehabilitation, may offer more significant advantages in promoting recovery across specific functional dimensions.

### 4.1 Effects of different rehabilitation interventions on lower limb function

This study found that intelligent rehabilitation (IR) ranked first in improving lower limb motor function, followed by aerobic rehabilitation (AR). This result aligns with conclusions from some previous traditional meta-analyses suggesting that exoskeleton robots and virtual reality technology benefit lower limb function in stroke patients (19, 20). However, this study further quantified the relative differences between different modalities through indirect comparisons. Concurrently, aerobic rehabilitation has been demonstrated to yield favorable outcomes in stroke and other neurological rehabilitation settings, further enhancing the credibility of the evidence (9, 21). Compared to previous reviews focusing on single intervention types, the observed advantages of IR may stem from its integrated benefits—including high repetitiveness, precise feedback, and task-oriented design—which likely contribute to these differences (22, 23). Moreover, high heterogeneity across studies (e.g., IR vs. SR, *I*^2^ = 91%) suggests that specific implementation details of smart rehabilitation may be critical variables influencing treatment outcomes.

### 4.2 Effects of different rehabilitation interventions on gait function

Neural regulation rehabilitation (NR) ranked highest in improving gait function, consistent with direct comparison results. This supports recent theories that non-invasive neural modulation can effectively regulate motor cortex excitability, thereby promoting walking function recovery (20, 24). This finding aligns with conclusions from meta-analyses examining neural modulation's impact on walking ability, enhancing the robustness of this evidence (25). Intelligent rehabilitation (IR) also demonstrated strong performance in gait function, resonating with lower limb function outcomes and further validating the comprehensive value of technology-assisted training in motor function rehabilitation. However, Traditional Chinese Medicine Rehabilitation (TCR) and Aerobic Rehabilitation (AR) ranked relatively lower in gait outcomes, differing from some studies focusing on the individual benefits of these therapies. This discrepancy may stem from the meta-analysis's synthesis of both direct and indirect comparisons, as well as variations in study populations, intervention doses, and outcome measurement tools across the included studies.

### 4.3 Heterogeneity treatment

This study acknowledges significant heterogeneity in certain comparisons, reflected in elevated *I*^2^ values (e.g., 82% *I*^2^ for NR vs. SR in gait function). The primary sources of this heterogeneity stem from clinical and methodological differences among included trials. Clinically, variations in patient characteristics—including chronic phase of stroke, baseline functional impairment, and presence of comorbidities—may influence treatment responsiveness. Methodologically, heterogeneity stemmed from variations in intervention protocols and differences in outcome assessment tools (e.g., diverse gait speed testing methods). Although a random-effects model was employed and funnel plots showed symmetrical distribution (indicating no significant publication bias), unexplained heterogeneity highlights study limitations. This underscores the need for cautious interpretation of treatment rankings and emphasizes the importance of conducting high-quality randomized controlled trials with standardized protocols in the future to reduce variability and strengthen evidence-based recommendations.

### 4.4 Limitations

This study has several limitations. First, comparisons of certain interventions—particularly those involving pairwise comparisons of TCR and IR components—are based on a limited number of trials, which may affect the reliability of ranking results. Second, significant heterogeneity exists across studies in terms of intervention protocols (e.g., intensity, duration, and specific techniques), patient characteristics (e.g., stroke phase and severity), and outcome measures, potentially impacting the comparability and generalizability of findings. Third, most rehabilitation trials had limitations in implementing blinding for participants and therapists, introducing potential risks of operational bias. Future research should prioritize high-quality head-to-head trials for underrepresented interventions, reduce heterogeneity through standardized protocols and outcome measures, and concurrently conduct cost-effectiveness analyses, long-term outcome studies, and real-world implementation research to facilitate evidence-based practice translation. Ultimately, advancing personalized rehabilitation strategies will be key to enhancing post-stroke care standards.

## 5 Conclusion

This study provides stratified evidence for selecting interventions to restore lower limb and gait function after stroke through a network meta-analysis. Results indicate that smart rehabilitation shows significant potential in improving lower limb function, while neuromodulation rehabilitation may offer greater advantages in enhancing gait function. In clinical practice, these top-ranked interventions should be prioritized based on patients' specific functional deficits, rehabilitation stage, and available medical resources. Future research should focus on conducting more high-quality, head-to-head randomized controlled trials, particularly for scarce interventions lacking direct comparisons. Additionally, further exploration is needed into efficacy differences across stroke subsubgroups and cost-effectiveness analyses to develop more personalized and actionable rehabilitation guidelines.

## Data Availability

The original contributions presented in the study are included in the article/Supplementary material, further inquiries can be directed to the corresponding authors.
