# Supplementary material for: Comparative effects of six rehabilitation therapies on lower limb function and gait function in stroke patients: a network meta-analysis of 33 RCTs
Source: Front Neurol. 2026 Mar 6;17:1759251. doi: 10.3389/fneur.2026.1759251 (PMC13002361; doi:10.3389/fneur.2026.1759251)
Supplement: Supplementary file 1 [file Table_1.docx]

Appendix 1: Basic Characteristics of Included Studies

| Included Studies | Country | Intervention Frequency and Duration | Disease Stage | Sample Size (Male/Female) | Age (mean±SD) | Intervention Category |
| --- | --- | --- | --- | --- | --- | --- |
| Arya 2019 [1] | India | 1h, 3-4 days/week, for 3 months | Chronic | E: 19 (15/4) C: 17 (15/2) | E: 48.16±8.36 C: 44.53±6.09 | Mirror Therapy |
| Bruno 2019 [2] | Brazil | 2 days/week, for 8 weeks | Chronic | E: 11 (5/6) C: 11 (4/7) | E: 66.4±10.1 C: 60.5±13.2 | Resistance Training |
| Bustamante Valles 2016 [3] | USA | 2h, 3-4 days/week, for 6-8 weeks | Chronic | E: 10 (3/7) C: 10 (4/6) | E: 44.1±12.55 C: 64.1±8.38 | Robotic-Assisted Training |
| Cui 2024 [4] | South Korea | 10min, 3 days/week, for 4 weeks (plus 30min conventional therapy) | Chronic | G1: 10 (7/3) G2: 10 (7/3) G3: 10 (6/4) | G1: 52.60±17.83 G2: 51.60±9.63 G3: 53.00±12.77 | Balance/Gaze Training |
| Dae-Hyouk 2013 [5] | South Korea | 40min, 5 days/week, for 4 weeks | Chronic | E: 15 (9/6) C: 15 (8/7) | E: 64.1±6.35 C: 58.9±7.03 | Action Observation Training |
| Fang 2024 [6] | China | 20min, 5 days/week, for 4 weeks | Acute | E: 18 (14/4) C: 18 (12/6) | E: 58.67±9.91 C: 57.00±12.61 | Neuromodulation + Gait Training |
| Hong 2024 [7] | China | E: Acupuncture 30min + Rehab 45min, 6 days/week, for 8 weeks C: Rehab 45min, 6 days/week, for 8 weeks | Acute | E: 30 (22/8) C: 30 (23/7) | E: 54.63±4.24 C: 55.00±3.79 | Acupuncture |
| Huo 2024 [8] | China | E: Robot 30min + Conventional, 5 days/week, for 4 weeks C: Conventional Training 30min + Conventional, 5 days/week, for 4 weeks | Subacute | E: 14 (10/4) C: 16 (11/5) | E: 57.93±11.47 C: 55.25±11.16 | Robotic-Assisted Training |
| Iqbal 2020 [9] | Pakistan | 40min, 4 days/week, for 4 weeks | Chronic | E: 32 (17/15) C: 32 (17/15) | E: 58.28±7.13 C: 58.87±6.13 | Dual-Task Training |
| Judit 2022 [10] | Hungary | 60min,5day/week，4 weeks | Chronic | 19 | U | aerobic exercise |
| Julie 2003 [11] | Canada | 30min, 3 days/week, until discharge | Acute | E: 68 (39/29) C: 65 (42/23) | E: 69.1±14.8 C: 72.0±12.1 | Resistance Training |
| Lee 2024 [12] | South Korea | 30min, 5 days/week, for 4 weeks (plus conventional therapy) | Acute/Chronic | E: 19 (10/9) C: 14 (11/3) | E: 63.79±11.76 C: 65.14±11.66 | Balance Training Device |
| Louie 2021 [12] | Canada | E: 60min, 3 days/week, for 8 weeks C: 45-60min, 4-5 days/week, for 8 weeks | Subacute | E: 19 (16/3) C: 17 (10/7) | E: 59.6±15.8 C: 55.3±10.6 | Exoskeleton Training |
| Louise 2024 [14] | UK | 2 weeks | Acute | E: 21 (14/7) C: 30 (15/15) | E: 73±12 C: 74±15 | Implicit Learning Approach |
| Mao 2022 [15] | China | 120min, daily, for 3 weeks | Chronic | E: 16 (13/3) C: 15 (12/3) | E: 52.25±9.21 C: 54.80±10.64 | Neuromuscular Electrical Stimulation |
| Marte 2008 [16] | Norway | 50min, 5 days/week, for 4 weeks | Subacute | E: 8 (3/5) C: 10 (4/6) | E: 60.8±13.0 C: 64.9±8.8 | Functional Strength Training |
| Michelle 2004 [17] | USA | 3 days/week, for 12 weeks | Chronic | E: 21 (U) C: 21 (U) | E: 65.8±11.5 C: 66.1±9.6 | Resistance Training |
| Moon 2022 [18] | South Korea | 30min, 3-5 days/week, for 4 weeks (plus conventional therapy) | Chronic | E: 12 (7/5) C: 12 (4/8) | E: 52.83±8.86 C: 57.75±10.95 | Action Observation Training |
| Rose 2011 [19] | USA | 1.5h, 5 days/week, for 3 weeks | Acute | E: 72 (31/41) C: 108 (45/63) | E: 67.6±15.3 C: 68.0±13.1 | Circuit Task Training |
| Sebastian 2020 [20] | USA | CARET: 40-60min, 3 days/week; CTI: 40min, 3 days/week; for 12 weeks | Chronic | E: 86 (60/26) C: 45 (21/24) | E: 59±11 C: 58±12 | Combined Aerobic, Resistance, and Cognitive Training |
| Shen 2023 [21] | China | 20min, 5 days/week, for 8 weeks (plus 30min conventional therapy) | Chronic | E: 20 (16/4) C: 20 (13/7) | E: 57.40±7.70 C: 56.10±7.99 | Dynamic Instability Training |
| Susan 2018 [22] | Canada | AT: 5 days/week; AT+RT: AT 3 days/week + RT 2 days/week, for 6 months | Chronic | AT: 35 (22/13) AT+RT: 33 (22/11) | AT: 65.6±13.2 AT+RT: 61.7±10.0 | Aerobic/Resistance Training |
| Susan 2021 [23] | USA | 90min, 3 days/week, for 8 weeks | Chronic | FE+RTP: 16 (12/4) VE+RTP: 14 (9/5) Control: 13 (12/1) | FE+RTP: 51±12 VE+RTP: 60±14 Control: 59±11 | Forced/Voluntary Aerobic Exercise |
| Tedla 2022 [24] | Saudi Arabia | 40-60min, 4 days/week, for 6 weeks | Subacute | G1: 18 (10/8) G2: 18 (9/9) G3: 18 (10/8) | G1: 58.78±5.46 G2: 57.83±6.72 G3: 58.50±6.42 | Neuromodulation |
| Valles 2016 [25] | USA | 2h, 3-4 days/week, for 6-8 weeks | Chronic | E: 10 (3/7) C: 10 (4/6) | E: 44.1±12.55 C: 64.1±8.38 | Robot-Assisted Circuit Training |
| Wu 2025 [26] | China | 5 days/week, for 4 weeks | Acute | E: 51 (33/18) C: 51 (36/15) | E: 62.24±9.78 C: 63.27±11.13 | Acupotomy |
| Zhang 2009 [27] | China | Rehabilitation: Once daily; Acupuncture+Rehab: Acupuncture 20min + Rehab, once daily; for 3 weeks | Acute | E: 40 (26/14) C: 40 (24/16) | E: 65.9±11.1 C: 69.2±9.7 | Acupuncture + Rehabilitation |
| Zhang 2013 [28] | China | IRT-TCM: 60min (30min Acupuncture + 30min Massage), once daily, for 21 days; Control: Conventional Rehab 60min, once daily, for 21 days | Acute | E: 42 (33/9) C: 19 (13/6) | E: 65.74±10.28 C: 66.95±10.95 | Integrated Traditional Chinese Medicine Rehabilitation |
| Zhang 2023 [29] | China | China | E: Mirror Therapy 30min, 1 time/day, for 3 months C: Conventional Rehab 30min, 2 times/day, for 3 months | Acute | E: 152 (79/73) C: 152 (78/74) | E: 57.46±3.85 C: 57.71±4.08 |
| Zhang 2024 [30] | China | 20min, 5 days/week, for 4 weeks | Subacute | E: 13 (10/3) C: 14 (11/3) | E: 56.15±10.43 C: 59.86±9.45 | Neuromodulation + Gait Training |
| Zhang 2024a [31] | China | E: Robot 60min + Conventional Rehab, 5 days/week, for 4 weeks C: Conventional Rehab 40min, 2 times/day, 5 days/week, for 4 weeks | Subacute | E: 12 (10/2) C: 12 (8/4) | E: 63.50±8.97 C: 63.83±8.28 | Robotic-Assisted Training |
| Zhu 2016 [32] | China | E: m-CIMT 120min + Conventional 45min, 5 days/week, for 4 weeks C: Conventional 45min, 5 days/week, for 4 weeks | Acute | E: 11 (U) C: 11 (U) | E: 59.18±7.34 C: 58.00±6.97 | Constraint-Induced Therapy |
| Zhu 2024 [33] | China | iTBS: 2 times/day, 5 days/week, for 2 weeks; PT: 60min, 5 days/week, for 2 weeks | Acute | E: 18 (14/4) C: 18 (13/5) | E: 58.67±7.24 C: 62.33±8.78 | Neuromodulation |

1. Arya, K. N., Pandian, S., & Kumar, V. (2019). Effect of activity-based mirror therapy on lower limb motor-recovery and gait in stroke: A randomised controlled trial. Neuropsychological rehabilitation, 29(8), 1193–1210. https://doi.org/10.1080/09602011.2017.1377087
2. Gambassi, B. B., Coelho-Junior, H. J., Paixão Dos Santos, C., de Oliveira Gonçalves, I., Mostarda, C. T., Marzetti, E., Sotão, S. S., Uchida, M. C., De Angelis, K., & Rodrigues, B. (2019). Dynamic Resistance Training Improves Cardiac Autonomic Modulation and Oxidative Stress Parameters in Chronic Stroke Survivors: A Randomized Controlled Trial. Oxidative medicine and cellular longevity, 2019, 5382843. https://doi.org/10.1155/2019/5382843
3. Bustamante Valles, K., Montes, S., Madrigal, M.deJ., Burciaga, A., Martínez, M. E., & Johnson, M. J. (2016). Technology-assisted stroke rehabilitation in Mexico: a pilot randomized trial comparing traditional therapy to circuit training in a Robot/technology-assisted therapy gym. Journal of neuroengineering and rehabilitation, 13(1), 83. https://doi.org/10.1186/s12984-016-0190-1
4. Cui, Z., Tang, Y. Y., Lee, M. H., & Kim, M. K. (2024). The effects of gaze stability exercises on balance, gait ability, and fall efficacy in patients with chronic stroke: A 2-week follow-up from a randomized controlled trial. Medicine, 103(32), e39221. <https://doi.org/10.1097/MD.0000000000039221>
5. Bang, D. H., Shin, W. S., Kim, S. Y., & Choi, J. D. (2013). The effects of action observational training on walking ability in chronic stroke patients: a double-blind randomized controlled trial. Clinical rehabilitation, 27(12), 1118–1125. https://doi.org/10.1177/0269215513501528
6. Fang, L., Zhang, W., Wu, J., Yu, H., Zhang, H., Chen, S., Zheng, B., Cao, M., Zhang, Y., Dai, L., & Chen, J. (2025). Effects of combining rTMs and augmented reality gait adaptive training on walking function of patients with stroke based on three-dimensional gait analysis and sEMG: a randomized controlled trial. Physiotherapy theory and practice, 41(5), 935–945. https://doi.org/10.1080/09593985.2024.2378905
7. Hong, X., Li, S., Zhong, Z., Lin, Y., & Zhang, K. (2024). Effects of acupuncture combined with trunk strengthening training on balance and gait abilities in stroke hemiplegic patients. Medicine, 103(29), e37784. https://doi.org/10.1097/MD.0000000000037784
8. Huo, C., Shao, G., Chen, T., Li, W., Wang, J., Xie, H., Wang, Y., Li, Z., Zheng, P., Li, L., & Li, L. (2024). Effectiveness of unilateral lower-limb exoskeleton robot on balance and gait recovery and neuroplasticity in patients with subacute stroke: a randomized controlled trial. Journal of neuroengineering and rehabilitation, 21(1), 213. https://doi.org/10.1186/s12984-024-01493-9
9. Iqbal, M., Arsh, A., Hammad, S. M., Haq, I. U., & Darain, H. (2020). Comparison of dual task specific training and conventional physical therapy in ambulation of hemiplegic stroke patients: A randomized controlled trial. JPMA. The Journal of the Pakistan Medical Association, 70(1), 7–10. https://doi.org/10.47391/JPMA.10443
10. Horváth, J., Debreceni Nagy, A., Fülöp, P., & Jenei, Z. (2022). Effectiveness of hospital-based low intensity and inspected aerobic training on functionality and cardiorespiratory fitness in unconditioned stroke patients: Importance of submaximal aerobic fitness markers. Medicine, 101(42), e31035. https://doi.org/10.1097/MD.0000000000031035
11. Moreland, J. D., Goldsmith, C. H., Huijbregts, M. P., Anderson, R. E., Prentice, D. M., Brunton, K. B., O'Brien, M. A., & Torresin, W. D. (2003). Progressive resistance strengthening exercises after stroke: a single-blind randomized controlled trial. Archives of physical medicine and rehabilitation, 84(10), 1433–1440. https://doi.org/10.1016/s0003-9993(03)00360-5
12. Lee, J. L., Min, J. H., Ko, S. H., Hwang, H., Baik, J. S., Park, I. J., Lee, Y. C., Shin, J. H., Hwang, K. J., Kim, C. H., & Shin, Y. I. (2025). Effectiveness of balance training using the balance pro (SBT-330) device in patients with reduced balance ability following a stroke: Randomized controlled trial. Journal of bodywork and movement therapies, 42, 381–386. https://doi.org/10.1016/j.jbmt.2024.12.020
13. Louie, D. R., Mortenson, W. B., Durocher, M., Schneeberg, A., Teasell, R., Yao, J., & Eng, J. J. (2021). Efficacy of an exoskeleton-based physical therapy program for non-ambulatory patients during subacute stroke rehabilitation: a randomized controlled trial. Journal of neuroengineering and rehabilitation, 18(1), 149. https://doi.org/10.1186/s12984-021-00942-z
14. Johnson, L., Burridge, J., Ewings, S., & Demain, S. (2024). A pilot cluster randomised controlled trial, of an IMPlicit learning approach versus standard care, on recovery of mobility following stroke (IMPS). Clinical rehabilitation, 38(10), 1346–1361. https://doi.org/10.1177/02692155241267205
15. Mao, Y. R., Zhao, J. L., Bian, M. J., Lo, W. L. A., Leng, Y., Bian, R. H., & Huang, D. F. (2022). Spatiotemporal, kinematic and kinetic assessment of the effects of a foot drop stimulator for home-based rehabilitation of patients with chronic stroke: a randomized clinical trial. Journal of neuroengineering and rehabilitation, 19(1), 56. https://doi.org/10.1186/s12984-022-01036-0
16. Bale, M., & Strand, L. I. (2008). Does functional strength training of the leg in subacute stroke improve physical performance? A pilot randomized controlled trial. Clinical rehabilitation, 22(10-11), 911–921. https://doi.org/10.1177/0269215508090092
17. Ouellette, M. M., LeBrasseur, N. K., Bean, J. F., Phillips, E., Stein, J., Frontera, W. R., & Fielding, R. A. (2004). High-intensity resistance training improves muscle strength, self-reported function, and disability in long-term stroke survivors. Stroke, 35(6), 1404–1409. https://doi.org/10.1161/01.STR.0000127785.73065.34
18. Moon, Y., & Bae, Y. (2022). The effect of backward walking observational training on gait parameters and balance in chronic stroke: randomized controlled study. European journal of physical and rehabilitation medicine, 58(1), 9–15. https://doi.org/10.23736/S1973-9087.21.06869-6
19. Rose, D., Paris, T., Crews, E., Wu, S. S., Sun, A., Behrman, A. L., & Duncan, P. (2011). Feasibility and effectiveness of circuit training in acute stroke rehabilitation. Neurorehabilitation and neural repair, 25(2), 140–148. https://doi.org/10.1177/1545968310384270
20. Koch, S., Tiozzo, E., Simonetto, M., Loewenstein, D., Wright, C. B., Dong, C., Bustillo, A., Perez-Pinzon, M., Dave, K. R., Gutierrez, C. M., Lewis, J. E., Flothmann, M., Mendoza-Puccini, M. C., Junco, B., Rodriguez, Z., Gomes-Osman, J., Rundek, T., & Sacco, R. L. (2020). Randomized Trial of Combined Aerobic, Resistance, and Cognitive Training to Improve Recovery From Stroke: Feasibility and Safety. Journal of the American Heart Association, 9(10), e015377. https://doi.org/10.1161/JAHA.119.015377
21. Shen, J., Ma, L., Gu, X., Fu, J., Yao, Y., Liu, J., & Li, Y. (2023). The effects of dynamic motion instability system training on motor function and balance after stroke: A randomized trial. NeuroRehabilitation, 53(1), 121–130. https://doi.org/10.3233/NRE-230008
22. Marzolini, S., Brooks, D., Oh, P., Jagroop, D., MacIntosh, B. J., Anderson, N. D., Alter, D., & Corbett, D. (2018). Aerobic With Resistance Training or Aerobic Training Alone Poststroke: A Secondary Analysis From a Randomized Clinical Trial. Neurorehabilitation and neural repair, 32(3), 209–222. https://doi.org/10.1177/1545968318765692
23. Linder, S. M., Davidson, S., Rosenfeldt, A., Lee, J., Koop, M. M., Bethoux, F., & Alberts, J. L. (2021). Forced and Voluntary Aerobic Cycling Interventions Improve Walking Capacity in Individuals With Chronic Stroke. Archives of physical medicine and rehabilitation, 102(1), 1–8. https://doi.org/10.1016/j.apmr.2020.08.006
24. Tedla, J. S., Rodrigues, E., Ferreira, A. S., Vicente, J., Reddy, R. S., Gular, K., Sangadala, D. R., Kakaraparthi, V. N., Asiri, F., Midde, A. K., & Dixit, S. (2022). Transcranial direct current stimulation combined with trunk-targeted, proprioceptive neuromuscular facilitation in subacute stroke: a randomized controlled trial. PeerJ, 10, e13329. https://doi.org/10.7717/peerj.13329
25. Bustamante Valles, K., Montes, S., Madrigal, M.deJ., Burciaga, A., Martínez, M. E., & Johnson, M. J. (2016). Technology-assisted stroke rehabilitation in Mexico: a pilot randomized trial comparing traditional therapy to circuit training in a Robot/technology-assisted therapy gym. Journal of neuroengineering and rehabilitation, 13(1), 83. https://doi.org/10.1186/s12984-016-0190-1
26. Wu, X. L., Lu, S. X., Wang, X. X., Dong, G. Q., Lu, M. Y., Zhang, Z. H., Sun, J. H., Hua, H. B., & Bai, L. J. (2025). Effect of ultrasound-guided acupotomy combined with acupuncture on limb dysfunction in patients with cerebral stroke. Neurological sciences : official journal of the Italian Neurological Society and of the Italian Society of Clinical Neurophysiology, 46(6), 2707–2716. https://doi.org/10.1007/s10072-025-08072-3
27. Zhang, N. X., Liu, G. Z., Huang, T. Q., Li, W. J., Luo, J. Q., Liu, W. W., Huang, Y., & Wang, A. M. (2009). Zhen ci yan jiu = Acupuncture research, 34(6), 406–409.
28. Zhang, Y., Jin, H., Ma, D., Fu, Y., Xie, Y., Li, Z., & Zou, Y. (2013). Efficacy of Integrated Rehabilitation Techniques of Traditional Chinese Medicine for ischemic stroke: a randomized controlled trial. The American journal of Chinese medicine, 41(5), 971–981. https://doi.org/10.1142/S0192415X13500651
29. Zhang, Y., Lyu, W., & Liu, J. (2023). Sichuan da xue xue bao. Yi xue ban = Journal of Sichuan University. Medical science edition, 54(5), 1046–1051. https://doi.org/10.12182/20230960602
30. Zhang, W., Dai, L., Fang, L., Zhang, H., Li, X., Hong, Y., Chen, S., Zhang, Y., Zheng, B., Wu, J., Cao, M., & Chen, J. (2024). Effectiveness of repetitive transcranial magnetic stimulation combined with intelligent Gait-Adaptability Training in improving lower limb function and brain symmetry after subacute stroke: a preliminary study. Journal of stroke and cerebrovascular diseases : the official journal of National Stroke Association, 33(12), 107961. https://doi.org/10.1016/j.jstrokecerebrovasdis.2024.107961
31. Zhang, Y., Zhao, W., Wan, C., Wu, X., Huang, J., Wang, X., Huang, G., Ding, W., Chen, Y., Yang, J., Su, B., Xu, Y., Zhou, Z., Zhang, X., Miao, F., Li, J., & Li, Y. (2024). Exoskeleton rehabilitation robot training for balance and lower limb function in sub-acute stroke patients: a pilot, randomized controlled trial. Journal of neuroengineering and rehabilitation, 21(1), 98. https://doi.org/10.1186/s12984-024-01391-0
32. Zhu, Y., Zhou, C., Liu, Y., Liu, J., Jin, J., Zhang, S., Bai, Y., Huang, D., Zhu, B., Xu, Y., & Wu, Y. (2016). Effects of modified constraint-induced movement therapy on the lower extremities in patients with stroke: a pilot study. Disability and rehabilitation, 38(19), 1893–1899. https://doi.org/10.3109/09638288.2015.1107775
33. Zhu, P. A., Li, Z. L., Lu, Q. Q., Nie, Y. Y., Liu, H., Kiernan, E., Yuan, J., Zhang, L. J., & Bao, X. (2024). Can cerebellar theta-burst stimulation improve balance function and gait in stroke patients? A randomized controlled trial. European journal of physical and rehabilitation medicine, 60(3), 391–399. https://doi.org/10.23736/S1973-9087.24.08307-2
