# Supplementary figures and images for: Comparative effects of six rehabilitation therapies on lower limb function and gait function in stroke patients: a network meta-analysis of 33 RCTs
Source: Front Neurol. 2026 Mar 6;17:1759251. doi: 10.3389/fneur.2026.1759251 (PMC13002361; doi:10.3389/fneur.2026.1759251)

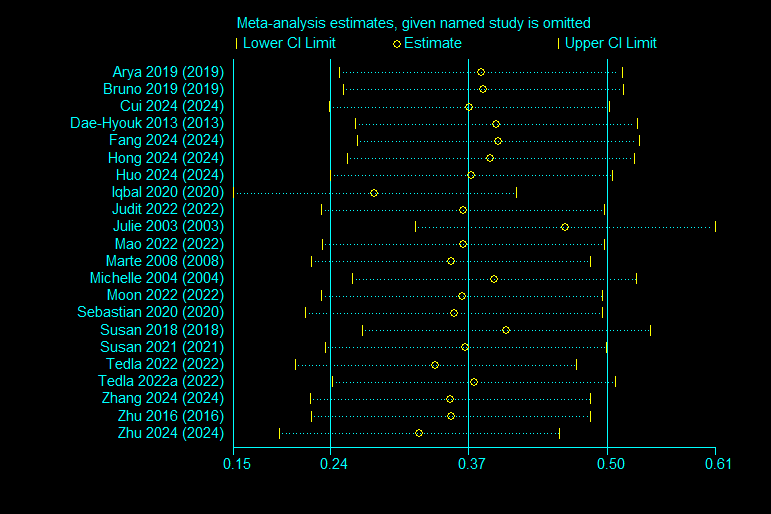


gait function


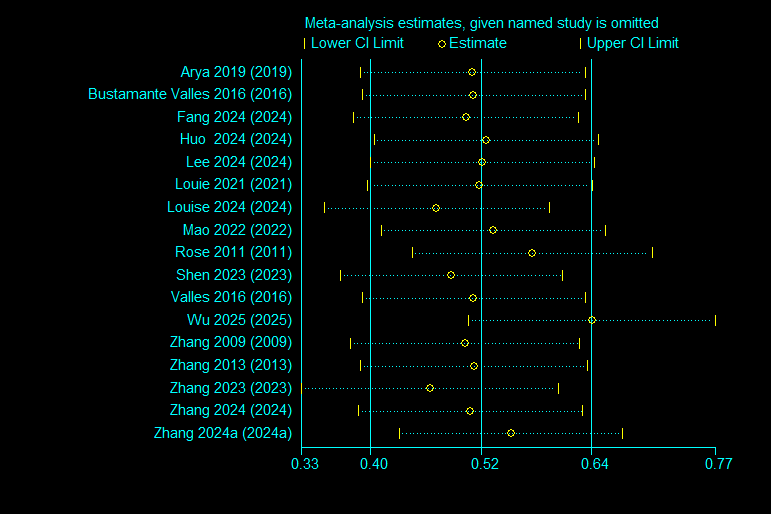


lower limb function

Supplement: Supplementary file 2 [file Table_2.docx]
